# Identification of key metabolic changes in renal interstitial fibrosis rats using metabonomics and pharmacology

**DOI:** 10.1038/srep27194

**Published:** 2016-06-03

**Authors:** Liangcai Zhao, Minjian Dong, Shixian Liao, Yao Du, Qi Zhou, Hong Zheng, Minjiang Chen, Jiansong Ji, Hongchang Gao

**Affiliations:** 1 School of Pharmaceutical Sciences, Wenzhou Medical University, Wenzhou, Zhejiang, China; 2 Lishui Central Hospital, the Fifth Affiliated Hospital, Wenzhou Medical University, Lishui, Zhejiang, China; 3 School & Hospital of Stomatology, Wenzhou Medical University, Wenzhou, Zhejiang, China

## Abstract

Renal fibrosis is one of the important pathways involved in end-stage renal failure. Investigating the metabolic changes in the progression of disease may enhance the understanding of its pathogenesis and therapeutic information. In this study, ^1^H-nuclear magnetic resonance (NMR)-based metabonomics was firstly used to screen the metabolic changes in urine and kidney tissues of renal interstitial fibrotic rats induced by unilateral ureteral obstruction (UUO), at 7, 14, 21, and 28 days after operation, respectively. The results revealed that reduced levels of bioenergy synthesis and branched chain amino acids (BCAAs), as well as elevated levels of indoxyl sulfate (IS) are involved in metabolic alterations of renal fibrosis rats. Next, by pharmacological treatment we found that reduction of IS levels could prevent the renal fibrotic symptoms. Therefore, we suggested that urinary IS may be used as a potential biomarker for the diagnosis of renal fibrosis, and a therapeutic target for drugs. Novel attempt combining metabonomics and pharmacology was established that have ability to provide more systematic diagnostic and therapeutic information of diseases.

Renal fibrosis is commonly companied by irreversible loss of kidney parenchyma and ultimately leads to destruction of the organizational structure, renal dysfunction, and even end-stage renal failure. The pathological mechanisms of renal fibrosis include disproportionate accumulation of extracellular matrix, proliferation of interstitial fibroblasts that replace kidney parenchyma, tubular dilation and atrophy, and infiltration of inflammatory cells resulting in tubular cell apoptosis and necrosis^1^,^2^,^3^. Understanding the pathogenesis of renal fibrosis holds great significance for the timely and accurate diagnosis and treatment of the disease. However, systemic and characteristic metabolic alterations involved in the pathological processes of renal fibrosis are still unclear^4^.

Metabonomics, a robust systemic biology approach used to identify global metabolic changes in biological systems, has been extensively used in the diagnosis and evaluation of diseases, provision of crucial insights into the pathogenesis of disease^5^,^6^. Using nuclear magnetic resonance (NMR)-based metabonomics, we comprehensively investigated the metabolic characteristics during diabetes evolution and found metabonomics was a valuable approach for providing novel insights into the pathogenesis of diabetes mellitus and its complications^7^,^8^,^9^. Korner *et al.* reported that urine composition in diabetes may result from the targeted effects of glucose on renal cells^10^. Williams *et al.* reported urinary metabolic profiles were found to be correlated with the animal development^11^. Zhao *et al.* identified perturbations of bile acid metabolism and phospholipid metabolism were closely related to chronic renal failure rats induced by adenine using metabonomics^12^. Then they screened potential biomarkers and drug targets involved in the disease^13^,^14^.

In our recent study, it was also shown that metabonomic analysis could be used as a tool to identify potential biomarkers and novel therapeutic targets involved in diabetic nephropathy mice^15^. We evaluated the patients’ recoverability of renal function after relief of obstruction from unilateral ureteral obstruction (UUO) by metabonomics, and found that this approach could help stratify patients, provide prognostic information, and be useful for postoperative surveillance^16^. We also studied the serum metabolic profiling of renal fibrotic rats induced by UUO^17^, and revealed enhanced pathways of lipid and ketone body synthesis, and disturbed tricarboxylic acid (TCA) cycle were partly contribute to the pathogenesis of renal fibrosis. Nevertheless, which metabolites and pathways are dominant during the pathogenic evolution of renal fibrosis needs further exploration.

To further understand the metabolic characteristics in renal fibrosis rats, time-related metabolic alterations in urine and renal tissue samples from UUO rats were determined holistically in comparison to the age-matched control rats in this study. Some crucial metabolites and pathways were identified by ^1^H NMR-based metabonomics, then a potential biomarker might be used as a diagnostic and therapeutic target for drugs were screened by pharmacology technologies.

## Methods

### Subjects and materials

ATP, valine, isoleucine, and leucine were obtained from Sigma-Aldrich Company. Meclofenamate was obtained from Cayman Chemical Company. Adult male Sprague-Dawley rats (age, 8 weeks; weight, 220 ± 15 g), obtained from Shanghai SLAC Laboratory Animal Company, China, were kept in specific pathogen-free colony of Laboratory Animal Center of Wenzhou Medical University, with regulated temperature and humidity and a 12 h light-dark cycle with lights switched on at 08:00 am. All animals received care and research procedures in accordance with the ARRIVE Guidelines^18^, which were also approved by the Institutional Animal Care and Use Committee of Wenzhou Medical University (document number: wydw2012-0083).

### UUO surgery and collection of samples

After acclimatization for 1 week in cages, the rats were randomly divided into sham-operated group (control group, n = 28) and UUO group (renal fibrosis group, n = 38). The UUO surgery and collection of samples were performed according to the operation procedure described earlier^4^. In brief, under chloral hydrate anesthesia, the left ureter of the rats was isolated and completely ligated using 4-0 silk suture. Control group rats underwent an identical surgical intervention except for ureteric ligation. The rats were weighed weekly and killed at 7, 14, 21, and 28 days after operation. Rats were allowed to recover from anesthesia and were housed in standard rodent cages (3–4 rats in each cage) with ad libitum access to water and food until sacrifice.

The urine samples were collected from 8 rats on the given day (8:00 pm–8:00 am). Samples with fecal contamination (0–2 per time point) were removed from subsequent analysis. Urine samples were centrifuged at 12,000 g for 10 min and the supernatant was stored at −80 °C until analysis. Under chloral hydrate anesthesia, the left kidney of the rats was immediately collected and divided into two portions by cross-cut. One half was fixed in 10% neutral buffered formalin and used for histological examination, and the other half was frozen at −80 °C for subsequent NMR studies.

### Histopathological examination

The formalin-fixed kidney tissues were embedded in paraffin and then cut into 4-μm sections. Kidney sections were stained using HE or periodic acid-Schiff, and tubulointerstitial damage was evaluated under light microscopy in whole kidney sections including cortex and outer medulla as described earlier^4^. Histopathological changes in kidney tissues were assessed in at least 25 randomly selected tissue sections from each group under study.

### ^1^H-NMR experiments of urine samples

The details for the preparation of samples and acquisition of ^1^H-NMR spectra refer to our previous studies^8^,^9^,^19^. Briefly, before NMR analysis, urine samples were thawed, and 200 μL of aliquots were mixed with 50 μL D_2_O and 300 μL of phosphate buffer (0.2 M Na_2_HPO_4_/NaH_2_PO_4_, pH 7.4) to minimize variations in pH. The mixtures were centrifuged to remove the precipitates, and then 500 μL of the supernatant was transferred to 5-mm NMR tubes. All NMR spectra were recorded at 25 °C on a Bruker AVANCE III 600 MHz NMR spectrometer equipped with a triple resonance probe and a z-axis pulsed field gradient. ^1^H-NMR spectra were acquired using a one-dimensional NOESY pulse sequence with water suppression during the relaxation delay of 4 s and a mixing time of 150 ms to avoid the rugged baseline resulted from some urine protein. 128 free induction decays were collected into 32 K data points with a spectral width of 12 000 Hz, an acquisition time of 2.66 s. FID was zero-filled to 64 K prior to Fourier transformation.

### ^1^H-NMR experiments of renal tissue extracts

The frozen renal tissue was weighed and put into a centrifuge tube. Ice-cold methanol (4 mL/g) and distilled water (0.85 mL/g) was added into the tube, homogenized at 4 °C after thawing and mixed by vortex. Chloroform (2 mL/g) and distilled water (2 mL/g) was added into the tube and mixed again. After the sample tubes kept on ice for 15 min, the homogenate was centrifuged at 1 000 g for 15 min at 4 °C. The supernatant was extracted and lyophilized for about 24 h. Before recording of the NMR spectra, the tissue extracts (small molecular weight metabolites) were resuspended in 500 μL D_2_O, and then the supernatant was transferred to 5 mm NMR tubes after centrifugation. ^1^H-NMR spectra were acquired at 25 °C on a Bruker AVANCE III 600 MHz NMR spectrometer equipped with a triple resonance probe and a z-axis pulsed field gradient. A one-dimensional ZGPR pulse sequence was used to achieve satisfactory water suppression in aqueous extracts. For each sample, 128 transients were collected into 64 K data points with a spectral width of 12 000 Hz and a relaxation delay of 6 s.

### Data reduction and multivariate pattern recognition analysis

All NMR spectra were phased and baseline corrected, and then data-reduced to 1100 integrated regions of 0.01 ppm width corresponding to the region of δ 10 to −1 using the Topspin 2.1 software package for multivariate pattern recognition analysis. And another data-reduced to 7334 integrated regions of 0.0015 ppm width corresponding to the region of δ 10 to −1 for quantitative analysis. For NMR spectra recorded in kidney extracts, the region of about δ 4.69 – 5.04 was removed to eliminate artifacts related to the residual water resonance. The region of the urine spectra associated with residual water and urea (4.71 – 5.06 and 5.72 – 5.94 ppm) were removed. The remaining spectral segments were normalized to the total sum of the spectral intensity to compensate for variations in total sample volume. The normalized integral values were then subjected to multivariate pattern recognition analysis using the SIMCA-P^+^ V12.0 software package (Umetrics, Umea, Sweden). Following a preliminary principal components analysis, supervised orthogonal partial least squares-discriminant analysis (OPLS-DA) was performed for class discrimination and biomarker identification^20^.

OPLS-DA was visualized using the first principal component (t[1]) and the orthogonal component (to[1]), which provided the most efficient 2D representation of the information, where the position of each point along a given axis in the scores plot was influenced by variables in the same axis in the loading plot. OPLS-DA revealed differences of different groups, which were necessary to eliminate outliers and enhance the quality of the model. The loading plots, which were assessed by the absolute value of the correlation coefficient, |r|, can identify the metabolites contribute to the separation of metabolic profiles^21^. The scores and loading plots complemented each other. A 100 random permutation test was also performed to evaluate the robustness of the OPLS-DA model. Meanwhile, two parameters were calculated: R^2^X and R^2^Y, the explained variance in the matrix of NMR data and class membership respectively, and Q^2^, the predictive capability of the model, which are commonly used to indicate the quality of model^22^. Values of R^2^Y and Q^2^ close to 1.0 represent an excellent model. Furthermore, the significance of the models was tested by CV-ANOVA in the SIMCA software^23^.

### Pharmacological experiments using exogenous metabolites or drugs

After acclimatization for 1 week prior to performing experiments, some other SD rats were operated under anesthesia as described earlier. After UUO treatment for 3 days, rats were randomly divided into five groups as follows: sham-operation control group, UUO model group, branched chain amino acids (BCAAs)-treated group (leucine/isoleucine/valine, *i.g.*, 300 mg/kg, q.d.)^24^, ATP-treated group (ATP, *i.v.*, 10 mg/kg, q.d.)^25^, meclofenamate-treated group (meclofenamate, *i.v.*, 10 mg/kg, q.d.)^26^. The clinical chemistry analysis of serum (n = 6–8 for each group at 7, 14 and 28 days after UUO) was performed for measuring biochemical parameters including serum creatinine, blood urea nitrogen (BUN) and choline using Automatic Analyzer (Beckman-Coulter LX-20).

### Statistical analysis

SPSS (Version 13.0 for Windows, SPSS Inc.) was used for statistical analysis. Independent samples t-test was used to detect significant differences in selected signals between different groups. *P* < 0.05 was considered to be statistically significant.

## Results

### Physiognomic and histopathological evaluation of UUO rats

**Figure 1:**
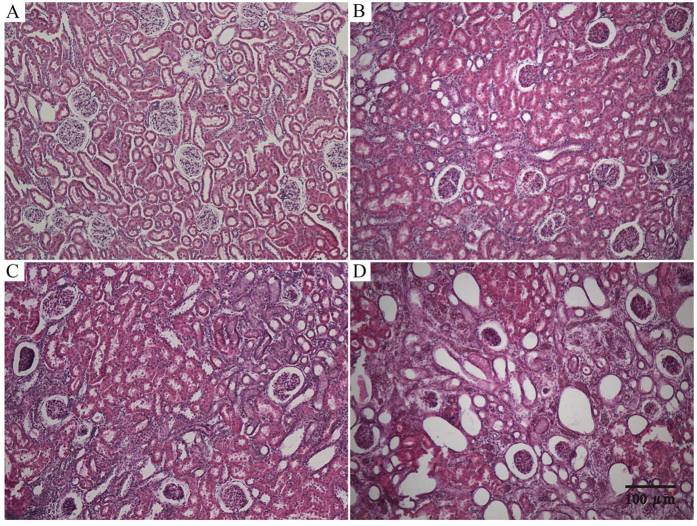
Changes in renal morphology in sham (**A**) and UUO-treated (**B**) for day 7, (**C**) for day 14 and (**D**) for day 28) kidneys as revealed by HE staining (100-fold).

All rats underwent UUO or sham operation fully recovered 24 h after surgery as assessed by ambulation, grooming, drinking and feeding. Compared to the nonligated contralateral kidney, it was found that the kidneys from the UUO ligated side were swollen, darker, turbid fluid, and significant renal parenchyma thinning. Microscopic examination showed the representative hematoxylin-eosin (HE)-stained sections of kidney from UUO and control rats (Fig. 1). No significant changes were observed in the tubulointerstitium of control kidneys, while UUO rats showed clear damages in a time-dependent manner. By day 7, significant tubulointerstitial injuries occurred including tubular dilation, atrophy, inflammatory cell infiltration, dilation of capillaries in the medulla, and an increase in interstitial fibroblasts. On postoperative days 14 and 28, more severe interstitial changes occurred including cortical thinning, further tubular dilation, renal papillary capillary expansion, inflammatory cell infiltration, increased number of fibroblasts, and interstitial expansion. Histopathological examination revealed that the histological severity of renal fibrotic rats was increasing after undergoing UUO.

### ^1^H-NMR based metabonomics of urine samples from UUO rats

**Figure 2:**
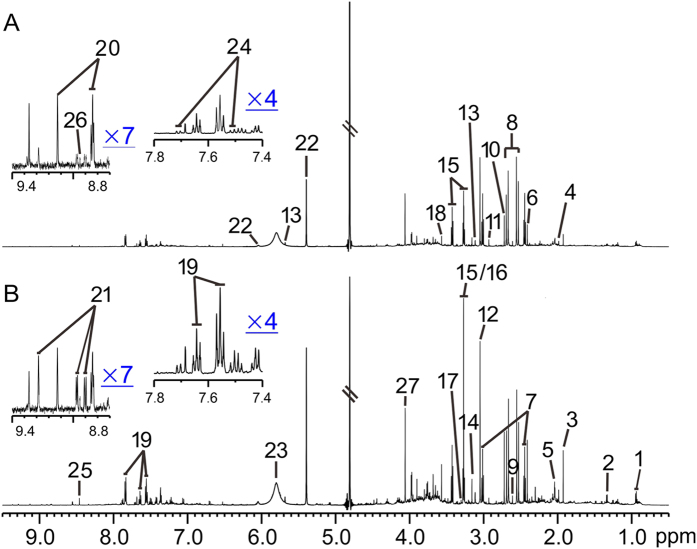
Representative ^1^H-NMR spectra of the urine samples obtained from a UUO rat (**A**) and a sham rat (**B**). Keys: 1, isoleucine/leucine; 2, lactate; 3, acetate; 4, acetamide; 5, N-acetylglycine; 6, succinate; 7, 2-ketoglutarate; 8, citrate; 9, methylamine; 10, dimethylamine; 11, trimethylamine; 12, creatinine/creatine; 13, cis-aconitate; 14, N-nitrosodimethylamine; 15, taurine; 16, trimethylamine-N-oxide; 17, trans-aconitate; 18, glycine; 19, hippurate; 20, trigonelline; 21, 1-methylnicotinamide (1-MNA); 22, allantoin; 23, urea; 24, indoxyl sulfate; 25, formate; 26, niacinamide; 27, creatinine.

**Figure 3:**
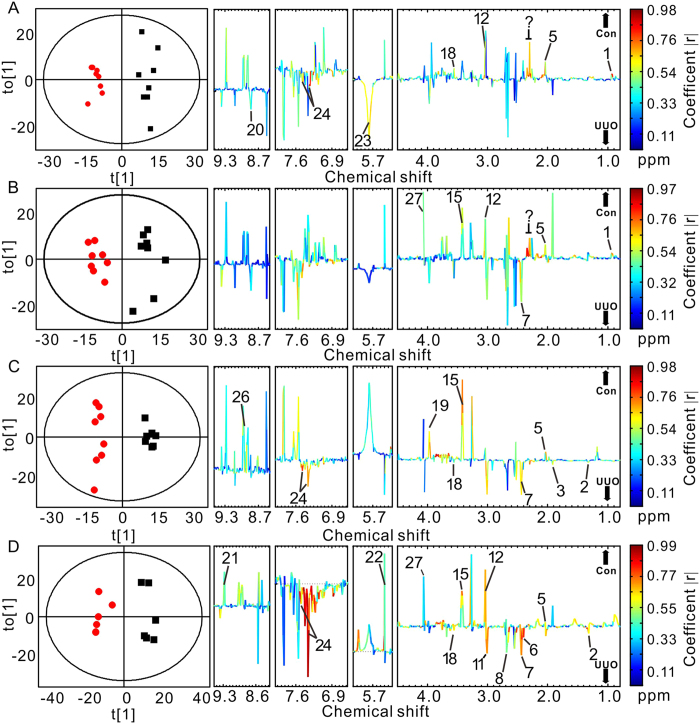
OPLS-DA scores (left) and coefficient-coded loading plots (right) for the models discriminating the UUO-treated groups (red dots) and time-matched control rats (blank squares) for data obtained from urine samples in 7 days (**A**), 14 days (**B**), 21 days (**C**), and 28 days (**D**) after UUO or sham operation. Peaks in the positive direction indicate metabolites that are more abundant in the control groups than UUO group (↑CON). Consequently, metabolites that are more abundant in the UUO group are presented as peaks in the negative direction (↓UUO). Metabolite keys are the same as in Fig. 2.

Representative ^1^H-NMR spectra of urine samples obtained from control and UUO-treated rats are shown in Fig. 2. Resonance assignments were attained based on our previous study^7^. To explore the metabolic profiles of renal fibrosis at different evolution stages, OPLS-DA was performed based on the ^1^H NMR spectra of the UUO 7 d, 14 d, 21 d and 28 d rats as well as their age-matched controls (Fig. 3). Clear discrimination along the direction of PC1 was shown between UUO rats and their controls at all the time points in the score plots (left). The parameters that evaluating the OPLS-DA model validity, R^2^Y, Q^2^ and p values for each model showed that the OPLS-DA models built were robust and credible (Table 1). The OPLS-DA loading plots suggests that the separation was attributed to the metabolites that have higher correlation (|r| > 0.55), including N-acetylglycine, creatinine/creatine, taurine, indoxyl sulfate (IS), lactate, glycine, myo-inositol, cis-aconitate, and etc.

Table 2 summarizes the |r| values of metabolites accounting for OPLS-DA separation models of urine samples and lists the results from the statistical analysis for comparison. The trend of the metabolite changes indicated by OPLS-DA loading plots was in consistent with that obtained by the statistical analysis. It was shown that UUO rats excreted lower levels of N-acetylglycine, creatinine/creatine, and taurine, with higher level in IS at most of the time points. Other metabolites, such as BCAAs (isoleucine/leucine) showed decreased in the urine samples of UUO rats at the early stage of renal fibrosis (postoperative 7 and 14 days); lactate, acetate were increased, with hippurate and niacinamide were decreased on postoperative 21 days; The tricarbosylic acid (TCA) cycle intermediates (2-ketoglutarate, citrate, succinate) were increased, with 1-methylnicotinamide (1-MNA) and allantoin were decreased on postoperative 28 days (Fig. S1). No statistically significant changes were found on methylamine and dimethylamine, which are closely related to methylamine metabolism.

### ^1^H-NMR based metabonomics of renal tissue extracts from UUO rats

**Figure 4:**
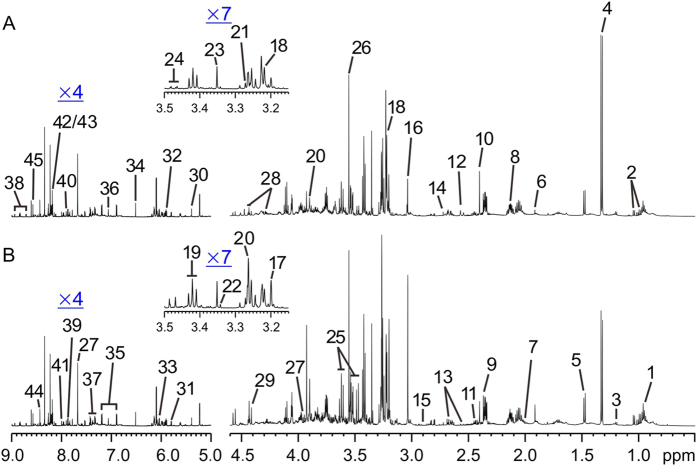
Representative ^1^H-NMR spectra of kidney extracts from rats in the UUO (**A**) and control (**B**) groups. Keys: 1, Isoleucine/Leucine; 2, Valine; 3, 3-Hydroxybutyrate; 4, Lactate; 5, Alanine; 6, Acetate; 7, Proline; 8, Glutamate; 9, Pyruvate; 10, Succinate; 11, Carnitine; 12, Methylamine; 13, Citrate; 14, Dimethylamine; 15, Trimethylamine (TMA); 16, Creatinine/Creatine; 17, Choline; 18, Phosphocholine/Glycerophosphosphorylcholine (PC/GPC); 19, Taurine; 20, Betaine; 21, Trimethylamine-*N*-oxide (TMAO); 22, 1, 3-Dimethylurate; 23, Methanol; 24, Glucose; 25, myo-Inositol; 26, Glycine; 27, Hippurate; 28, myo-Inosine; 29, Trigonelline; 30, Allantion; 31, Uracil; 32, cis-Aconitate; 33, Adenosine; 34, Fumarate; 35, Tyrosine; 36, Histidine; 37, Phenylalanine; 38, Niacinamide; 39, Histamine; 40, Xanthine; 41, 3-Methylxanthine; 42, Hypoxanthine; 43, Adenine; 44, Formate; 45, AMP/ATP.

**Figure 5:**
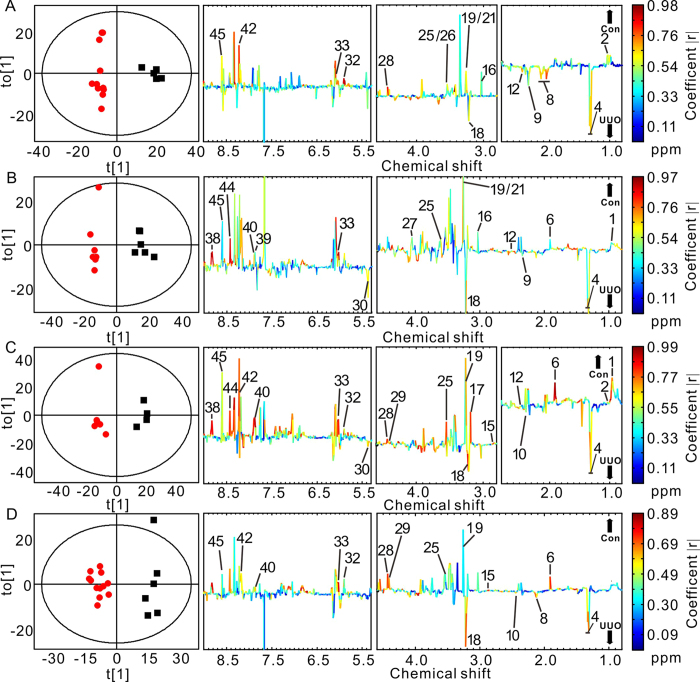
Multivariate pattern recognition analysis of kidney extracts from UUO-treated and control rats at different time points. OPLS-DA scores (left) and coefficient-coded loading plots (right) for the models discriminating the UUO-treated group (red dots) and time-matched control group (blank squares) for data obtained from kidney extracts in 7 days (**A**), 14 days (**B**), 21 days (**C**), and 28 days (**D**) after UUO or sham surgery. Peaks in the positive direction indicate metabolites that are more abundant in the control groups than UUO group (↑CON). Consequently, metabolites that are more abundant in the UUO group are presented as peaks in the negative direction (↓UUO). Metabolite keys are the same as in Fig. 4.

Figure 4 shows representative ^1^H-NMR spectra of the renal tissue extracts obtained from UUO-treated and control rats, respectively, along with the resonance assignments of the metabolites attained based on our recent study^15^. Similar to the metabonomics data of urine samples, the OPLS-DA score plots of kidney extracts showed distinct, easily detectable differences between the UUO rats and their controls (Fig. 5 left, Table 1). The metabolites that have high |r| values illustrated by loading plots (Fig. 5 right) and statistical analysis (Table 3) revealed that UUO rats exhibited progressively lower levels of acetate, taurine, myo-inositol, cis-aconitate, adenosine, and AMP/ATP, with higher levels in lactate and PC/GPC at all of the studied time points. Besides, pyruvate was increased with creatinine/creatine and TMAO were decreased at the early stage of renal fibrosis (postoperative 7 and 14 days); isoleucine/leucine, niacinamide and histamine were decreased, with allantoin was increased on postoperative 14 and 21 days; choline, trigonelline, and hypoxanthine were decreased, with succinate was increased at the later stage of renal fibrosis (postoperative 21 and 28 days, Fig. S2). These might be crucial metabolites associated with the mechanisms underlying the initiation and progression of renal fibrosis.

### Identification of dominant metabolites involved in renal fibrosis

**Figure 6:**
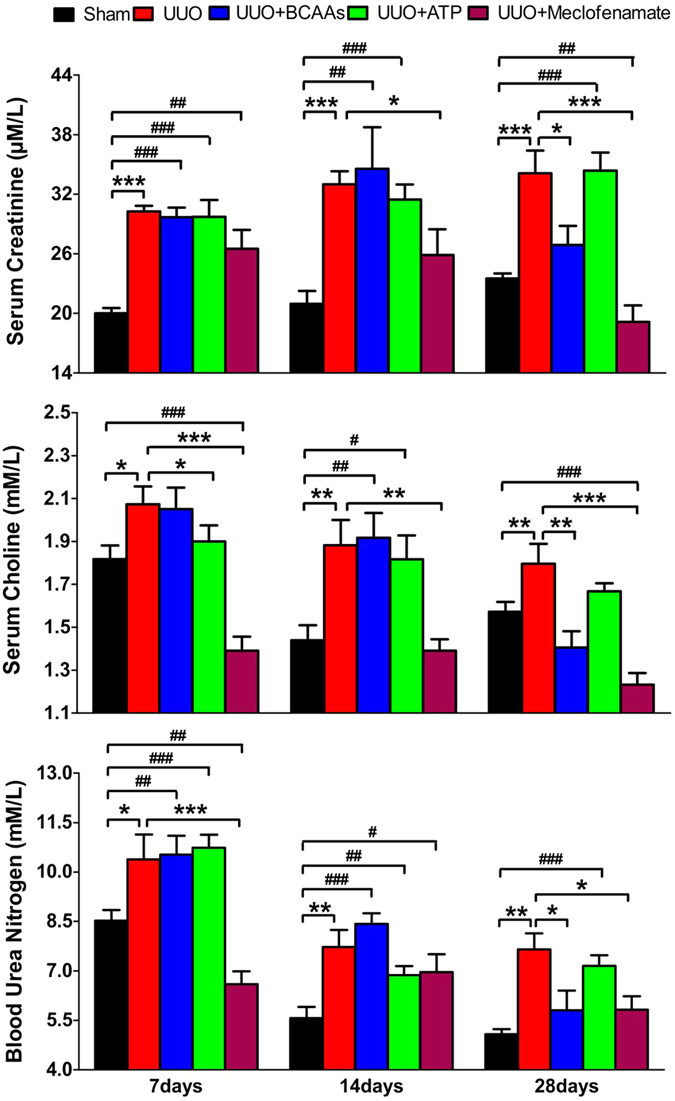
Serum clinical chemistry parameters at different days after administration of BCAAs, ATP and meclofenamate, respectively. Keys: ^*^P < 0.05, ^**^P < 0.01, ^***^P < 0.001, compared with UUO rats, ^#^P < 0.05, ^##^P < 0.01, ^###^P < 0.001, compared with sham operation rats.

According to the change trends of metabolites, we accordingly complemented the levels of BCAAs and ATP as well as the administration of an IS antagonist^26^, meclofenamate in UUO rats. The clinical blood biochemical parameters including BUN, creatinine, and choline treated with exogenous drugs are presented in Fig. 6. Compared with UUO rats, these parameters recovered to some extent at three studied time points (7, 14 and 28 days). In meclofenamate-treated UUO rats, almost all the parameters recovered to normal at three time points, indicating that renal function could be ameliorated after administration of meclofenamate. In contrast, ATP treatment had almost no effects on the amelioration of blood biochemical parameters. After administration of BCAAs, these parameters recovered to normal only at the time point of 28 days, suggesting that BCAAs could partially improve the renal fibrotic symptoms. It should be mentioned that UUO rats treated with meclofenamate seem to have lower creatinine, choline and BUN compared to controls. We suspected that meclofenamate might modify the creatinine, choline and BUN metabolism of UUO rats.

**Figure 7:**
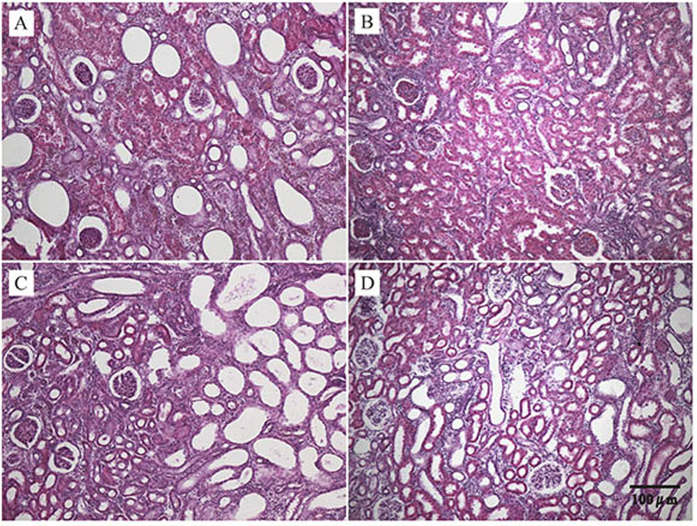
Representative HE-stained sections (100-fold) of kidneys from UUO (**A**), and BCAAs-treated UUO rats (**B**), ATP-treated UUO rats (**C**), and meclofenamate-treated UUO rats (**D**) for 28 days, respectively.

Figure 7 shows representative images of HE-stained sections of kidney tissues from UUO rats after administration of different agents at the time points of 28 days. It was found that after 28 days of drug administration, both meclofenamate and BCAAs treatment significantly attenuated tubular damages observed in renal fibrotic rats, with less signs of tubular denaturation or necrosis. Moreover, Masson-staining also showed clear renal fibrosis and thickening of Bowman’s capsule in kidneys of UUO rats, which were obviously ameliorated by meclofenamate or BCAAs treatment, while not by ATP (Fig. S3).

## Discussion

Renal interstitial fibrosis is a dynamic system that involves consecutive events including apoptosis, necrosis, and proliferation in local renal tissues^27^. UUO in the rodents is a classic model of renal interstitial fibrosis that is similar in many aspects to patients with renal fibrosis observed clinically^4^,^28^,^29^. The holistic metabolic changes described by NMR-based metabonomics were in good agreement with the hematological and histopathological results. Combining metabonomics and pharmacology, some crucial metabolites and pathways of renal fibrosis were identified and a potential biomarker might be used as a diagnostic and therapeutic target for drugs were screened. To our knowledge, this joint approach is a novel attempt to provide more systematic diagnostic and therapeutic information of diseases.

### Kidney dysfunction in UUO rats

The reduced urinary levels of creatinine detected at all the time points studied in this work might be due to a variety of factors including creatinine reabsorption, cell leakage, and changes in both muscle mass and caloric intake^30^. Depletion of creatinine was also observed in diabetic nephropathy rats with altered renal tubular function and morphology^31^. Furthermore, as urinary allantoin was not reabsorbed across the proximal tubule, the reduction in urinary allantoin excretion detected in the UUO rats is indicative of reduced glomerular filtration rate. These results are consistent with the changes in renal morphology and associated with the onset of renal dysfunction and renal fibrosis of UUO rats.

### Metabonomics of metabolic pathways altered in UUO rats

**Figure 8:**
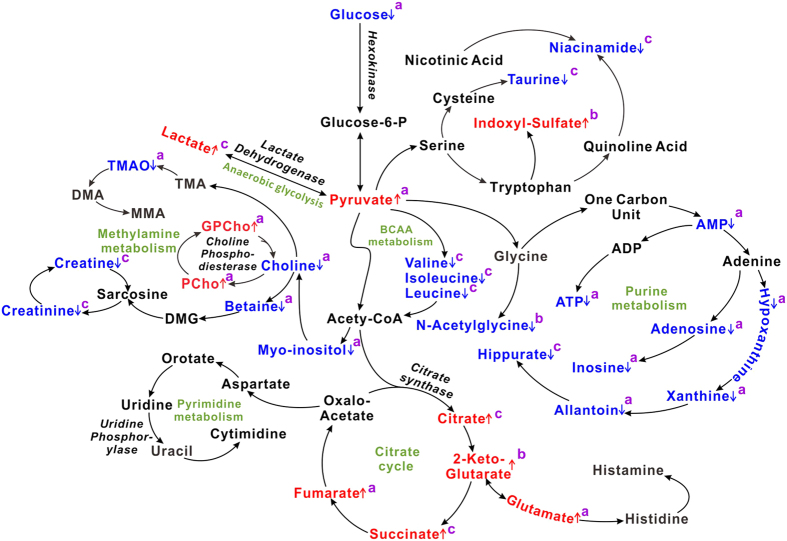
Altered metabolic pathways related to renal fibrosis induced by UUO. The pathways referenced to the KEGG database and Roche Biochemical Pathways Part 1 (website: http://biochemical-pathways.com/#/map/1) show the interrelationship of the identified metabolic pathways involved in UUO rats. The metabolites with the color blue or red are representative of decline and elevation in levels, respectively, compared to controls. Keys: ^a^detected in kidney; ^b^detected in urine; ^c^detected in both kidney and urine.

Metabonomics revealed that during the initiation and progression of renal fibrosis of UUO rats, the changes of metabolic pathways including TCA cycle, anaerobic glycolysis, amino acid metabolism (such as BCAAs and tryptophan), purine metabolism and methylamine metabolism (Fig. 8). Creatinine, a marker of renal dysfunction, showed decreased in urine and kidney, which directly suggest the onset of renal fibrosis. Methylamine, dimethylamine, and trimethylamine, the important metabolites in methylamine metabolism, appeared low correlations |r| in the loading plot, which suggests that methylamine metabolism have not much contribution to the urinary metabolic alterations of UUO rats. The elevated levels of IS in urinary samples of UUO rats suggest enhanced tryptophan metabolism during the onset and evolution of renal fibrosis. Conversely, decreased levels of valine, leucine, and isoleucine indicated the synthesis of BCAAs was inhibited in UUO rats. Also lower levels of AXP, adenosine, inosine, and xanthine indicate reduced purine metabolism and bioenergy production in UUO-induced renal fibrosis rats.

Glucose metabolism plays an important role in the evolution of renal fibrosis^17^. In this study, consecutively increased levels of TCA cycle intermediates (such as citrate, succinate and 2-ketoglutarate) and anaerobic glycolysis products (pyruvate and lactate) suggest that TCA cycle and anaerobic glycolysis are strengthened in UUO-treated rats. The lower glucose level in renal tissue extracts confirms that glucose metabolism is upregulated during renal fibrotic process. Interestingly, enhanced pathways of metabolism of lipids and ketone bodies in serum of UUO-treated rats were found in our previous study^17^, which suggests that the cells switch to glucose and lipid metabolism to maintain energy homeostasis during the progression of renal fibrosis.

### Dominant metabolic changes in UUO rats

**Figure 9:**
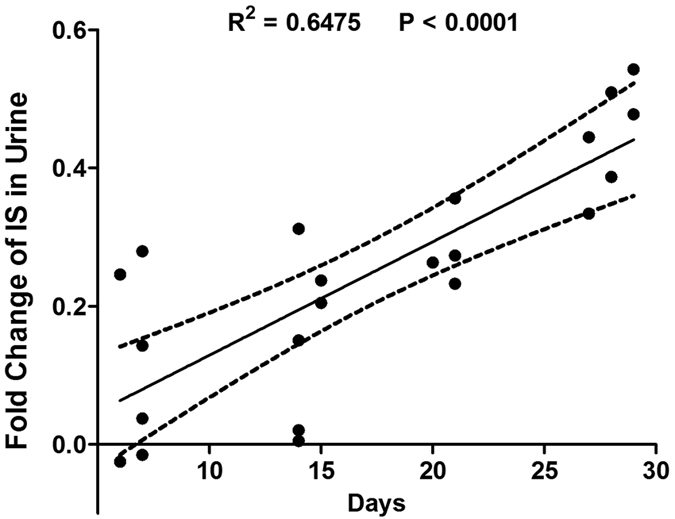
Linear regression analysis of urinary IS levels in UUO rats. The y-axis is fold changes of IS in urine [(UUO-control)/control]. IS levels were increased at all the studied time points (r^2^ = 0.6475, *P* < 0.0001).

Among all metabolites changed in UUO rats, it is worth noting that urinary IS appeared a constant increased trend at all time points in this study. IS, as one of representative sulfate-conjugated metabolites, is excreted in the urine under normal renal function^32^, while an increase in urinary IS level was found under oxidative stress due to its cytotoxic effect^33^,^34^. Therefore we speculate that IS may be a potential biomarker for the diagnosis of renal fibrosis. For further confirmation, we carried out the pharmacological experiments. As expected, the biochemical and histopathological results showed that UUO rats treated with meclofenamate were recovered to the normal pattern at all time points, suggesting that IS antagonist may effectively improve the renal fibrotic symptoms. However, administrations of ATP were not effective for the treatment of renal fibrosis. In addition, we also found a significant positive association between the rate of change in IS level and the time after UUO operation (r^2^ = 0.6475, *P* < 0.0001, Fig. 9). It was also reported that IS level was elevated in patients with chronic kidney disease (CKD) and was closely correlated with the progression of CKD in serum^35^. Therefore, we hypothesize that urinary IS may be used as a potential biomarker for the diagnosis and therapeutic target of renal fibrosis.

In this study, we also found that the metabolism of BCAAs was closely related to renal fibrosis. Decreased levels of the BCAAs have also been reported in patients with hemodialysis^36^ and with acute or chronic uremia^37^,^38^,^39^. BCAAs are found in dietary proteins, and hence the decrease in urine and kidney BCAA levels observed in the present study might result from reduction of dietary BCAAs intake^40^. BCAAs play important roles in maintenance and growth of skeletal muscle, which can be used as an energy source during exercise, and serve as gluconeogenic precursors^40^,^41^. And thus, BCAAs-supplemented mice were accompanied by increased mitochondrial biogenesis and function in skeletal muscles^41^, which may partly contribute to the less signs of tubular denaturation or necrosis detected from HE-stained sections of UUO kidney tissues after BCAAs treatment studied in the present study (Fig. 7). In patients with decompensated liver cirrhosis, Kawamura *et al.* reported that oral BCAAs supplementation restored serum bilirubin and albumin levels^42^, and absolute lymphocyte count^43^. Despite a better understanding of biological properties of BCAAs in animals and in patients, the beneficial roles and mechanisms of BCAAs on renal fibrosis remains not fully elucidated. This study suggests that the early supplementation of BCAAs through dietary should be expected to ameliorate and postpone the renal fibrosis.

In conclusion, the joint approach combining metabonomics and pharmacology provides a novel promising technique to elucidate the mechanisms of renal fibrosis. A few of metabolites and their metabolic pathway including TCA cycle, anaerobic glycolysis, and amino acid metabolism were found closely related to the initiation and progression of renal fibrosis. Urinary IS may be used as a potential biomarker for the diagnosis and therapeutic target of renal fibrosis.

## Additional Information

**How to cite this article**: Zhao, L. *et al.* Identification of key metabolic changes in renal interstitial fibrosis rats using metabonomics and pharmacology. *Sci. Rep.* **6**, 27194; doi: 10.1038/srep27194 (2016).

## Supplementary Material

Supplementary Information

## Figures and Tables

**Table 1:** Cross-validated data of OPLS-DA models in UUO rats compared to age-matched controls.

Model	Components	R^2^X	R^2^Y	Q^2^	P
Urine 7 days	1 + 1	0.28	0.96	0.62	0.02
Urine 14 days	1 + 2	0.50	0.98	0.81	0.01
Urine 21 days	1 + 2	0.46	0.98	0.78	0.02
Urine 28 days	1 + 1	0.42	0.95	0.73	0.03
kidney 7 days	1 + 2	0.54	0.97	0.77	0.02
kidney 14 days	1 + 1	0.43	0.97	0.73	0.02
kidney 21 days	1 + 1	0.56	0.97	0.80	0.05
kidney 28 days	1 + 1	0.41	0.91	0.63	0.03

**Table 2:** |r| values of metabolites accounting for OPLS-DA models of urine samples and statistical analysis for comparison between UUO and control rats.

No	Metabolites	7 Days UUO versus control		14 Days UUO versus control		21 Days UUO versus control		28 Days UUO versus control	
		r value	Fold change	r value	Fold change	r value	Fold change	r value	Fold change
1	Isoleucine/leucine	0.77	−0.14^**^	0.73	−0.17^*^	0.32	+0.08	0.23	−0.08
2	Lactate	0.20	−0.08	0.25	−0.04	0.57	+0.39^*^	0.62	+0.65^*^
3	Acetate	0.11	+0.25	0.34	−0.35	0.53	+0.77^*^	0.20	−0.31
4	Acetamide	0.33	+0.00	0.30	+0.04	0.35	−0.06	0.48	+0.01
5	N-Acetylglycine	0.79	−0.24^**^	0.76	−0.25^**^	0.74	−0.18^**^	0.53	−0.15^*^
6	Succinate	0.33	−0.20	0.01	+0.02	0.05	+0.25	0.58	+1.42^*^
7	2-Ketoglutarate	0.02	+0.05	0.49	+0.47^*^	0.59	+0.40^*^	0.67	+0.71^*^
8	Citrate	0.19	+0.12	0.40	+0.24	0.21	+0.27	0.69	+1.28^**^
9	Methylamine	0.05	−0.05	0.43	+0.20	0.16	+0.09	0.42	+0.08
10	Dimethylamine	0.08	−0.01	0.29	+0.04	0.21	+0.15	0.31	+0.01
11	Trimethylamine	0.16	+0.09	0.24	+0.27	0.26	+0.37	0.66	+0.25^*^
12	Creatinine/Creatine	0.63	−0.10^*^	0.65	−0.23^*^	0.47	−0.06	0.73	−0.26^*^
13	cis-Aconitate	0.31	+0.12	0.23	−0.01	0.06	−0.09	0.28	−0.10
15	Taurine	0.21	−0.15	0.53	−0.23^*^	0.66	−0.23^*^	0.64	−0.34^**^
17	trans-Aconitate	0.19	+0.06	0.34	+0.18	0.06	+0.05	0.10	+0.02
18	Glycine	0.58	−0.23^*^	0.47	+0.14	0.46	+0.29^*^	0.63	+0.54^*^
19	Hippurate	0.51	+0.19	0.39	+0.31	0.66	−0.21^*^	0.26	−0.04
20	Trigonelline	0.47	+0.28	0.28	+0.42	0.16	+0.00	0.15	+0.01
21	1 − MNA	0.24	−0.33	0.24	−0.27	0.40	−0.30	0.61	−0.48^*^
22	Allantoin	0.42	−0.08	0.45	−0.10	0.53	+0.04	0.64	−0.18^*^
23	Urea	0.55	+0.09	0.17	+0.02	0.35	−0.09	0.38	−0.20
24	Indoxyl sulfate	0.56	+0.20^*^	0.30	+0.29	0.66	+0.15^*^	0.79	+0.45^**^
26	Niacinamide	0.36	−0.19	0.53	−0.32	0.69	−0.29^*^	0.48	−0.22
27	Creatinine	0.25	−0.05	0.60	−0.18^*^	0.09	−0.07	0.65	−0.25^*^

Key: 1-MNA, 1-Methylnicotinamide; UUO, unilateral ureteral obstruction.

**Table 3:** |r| values of metabolites accounting for OPLS-DA models of renal extracts and statistical analysis for comparison between UUO and control rats.

Key	Metabolites	7 Days UUO versus control		14 Days UUO versus control		21 Days UUO versus control		28 Days UUO versus control	
		r value	Fold change	r value	Fold change	r value	Fold change	r value	Fold change
1	Isoleucine/leucine	0.11	−0.08	0.66	−0.30^*^	0.67	−0.37^*^	0.22	−0.13
2	Valine	0.61	−0.23^*^	0.20	−0.24	0.68	−0.28^*^	0.09	−0.16
4	Lactate	0.74	+0.38^**^	0.70	+0.53^**^	0.70	+0.45^**^	0.63	+0.52^**^
6	Acetate	0.49	−0.10	0.65	−0.21^**^	0.94	−0.24^*^	0.84	−0.25^**^
8	Glutamate	0.56	+0.35^*^	0.42	+0.09	0.13	+0.04	0.72	+0.23^*^
9	Pyruvate	0.53	+0.35^*^	0.50	+0.13^*^	0.20	+0.17	0.12	+0.09
10	Succinate	0.49	+0.19	0.46	+0.11	0.91	+0.19^*^	0.71	+0.18^*^
12	Methylamine	0.58	+0.44^*^	0.75	−0.09^*^	0.66	−0.20^*^	0.27	−0.11
13	Citrate	0.46	+0.14	0.43	+0.15	0.46	+0.14	0.50	+0.20
15	Trimethylamine	0.44	+0.05	0.24	−0.17	0.69	−0.23^*^	0.60	−0.12^*^
16	Creatinine/Creatine	0.65	−0.17^*^	0.59	−0.15^*^	0.51	+0.05	0.35	−0.08
17	Choline	0.35	−0.05	0.52	−0.10	0.79	−0.21^*^	0.38	−0.19
18	PC/GPC	0.42	+0.49^**^	0.57	+0.85^**^	0.90	+0.65^**^	0.56	+0.66^**^
19	Taurine	0.57	−0.24^*^	0.59	−0.23^**^	0.76	−0.34^**^	0.34	−0.17^*^
21	TMAO	0.56	−0.24^*^	0.51	−0.29^**^	0.49	−0.02	0.33	−0.11
24	Glucose	0.21	+0.01	0.34	−0.24	0.42	−0.11	0.35	−0.09
25	myo-Inositol	0.58	−0.27^*^	0.57	−0.49^*^	0.83	−0.16^*^	0.44	−0.38^**^
26	Glycine	0.38	−0.16^*^	0.32	−0.06	0.30	−0.23	0.25	−0.23
27	Hippurate	0.04	−0.08	0.56	−0.15^*^	0.54	−0.01	0.05	−0.09
28	myo-Inosine	0.80	−0.07^*^	0.40	−0.09	0.73	−0.24^**^	0.66	−0.17^**^
29	Trigonelline	0.40	−0.03	0.16	−0.06	0.57	−0.31^**^	0.64	−0.13^*^
30	Allantoin	0.43	+0.03	0.59	+0.14^*^	0.61	+0.49^*^	0.01	−0.05
31	Uracil	0.45	+0.48	0.27	+0.18	0.40	−0.12	0.33	+0.03
32	cis-Aconitate	0.94	−0.60^**^	0.34	+0.07	0.96	−0.68^**^	0.63	−0.55^**^
33	Adenosine	0.68	−0.48^**^	0.97	−0.37^*^	0.95	−0.52^**^	0.75	−0.31^*^
34	Fumarate	0.41	+0.70	0.13	+0.38	0.03	−0.37	0.35	+0.05
36	Histidine	0.26	+0.18	0.09	+0.17	0.34	+0.20	0.35	+0.71
38	Niacinamide	0.52	−0.26	0.95	−0.45^*^	0.93	−0.15^**^	0.63	−0.24
39	Histamine	0.26	+0.24	0.63	−0.37^*^	0.19	+0.12	0.32	+0.22
40	Xanthine	0.02	+0.24	0.59	−0.13^*^	0.85	−0.62^**^	0.50	−0.11^*^
42	Hypoxanthine	0.72	−0.13^*^	0.51	−0.21	0.79	−0.50^**^	0.55	−0.22^*^
44	Formate	0.45	−0.06	0.96	−0.27^*^	0.93	−0.10^**^	0.47	−0.01
45	AMP/ATP	0.56	−0.38^*^	0.40	−0.84^**^	0.59	−0.85^**^	0.41	−0.73^**^

Key: PC/GPC: Phosphocholine/Glycerophosphosphorylcholine, TMAO: Trimethylamine-*N*-oxide.
