# Supplementary material for: Identification of key metabolic changes in renal interstitial fibrosis rats using metabonomics and pharmacology
Source: Sci Rep. 2016 Jun 3;6:27194. doi: 10.1038/srep27194 (PMC4891668; doi:10.1038/srep27194)
Supplement: Supplementary Information [file srep27194-s1.pdf]

Identification of key metabolic changes in renal interstitial fibrosis rats using metabonomics and pharmacology

Liangcai Zhao, Minjian Dong, Shixian Liao, Yao Du, Qi Zhou, Hong Zheng, Minjiang Chen, Jiansong Ji, & Hongchang Gao

Supplement information:

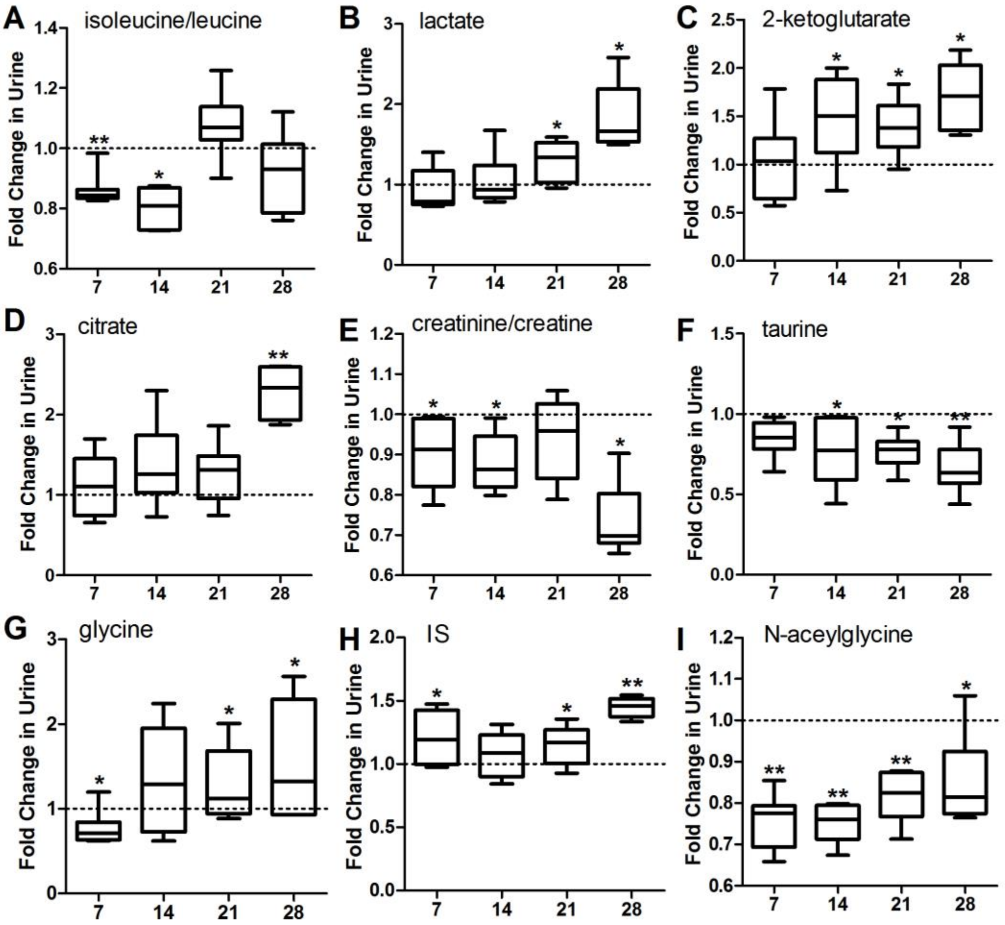

Figure S1 | Relative abundances of metabolites obtained from ^{1}H NMR spectra of urine samples collected from the UUO rats compared to the age-matched control rats at the different time points after UUO or sham surgery. Keys: IS, indoxylsulfate.

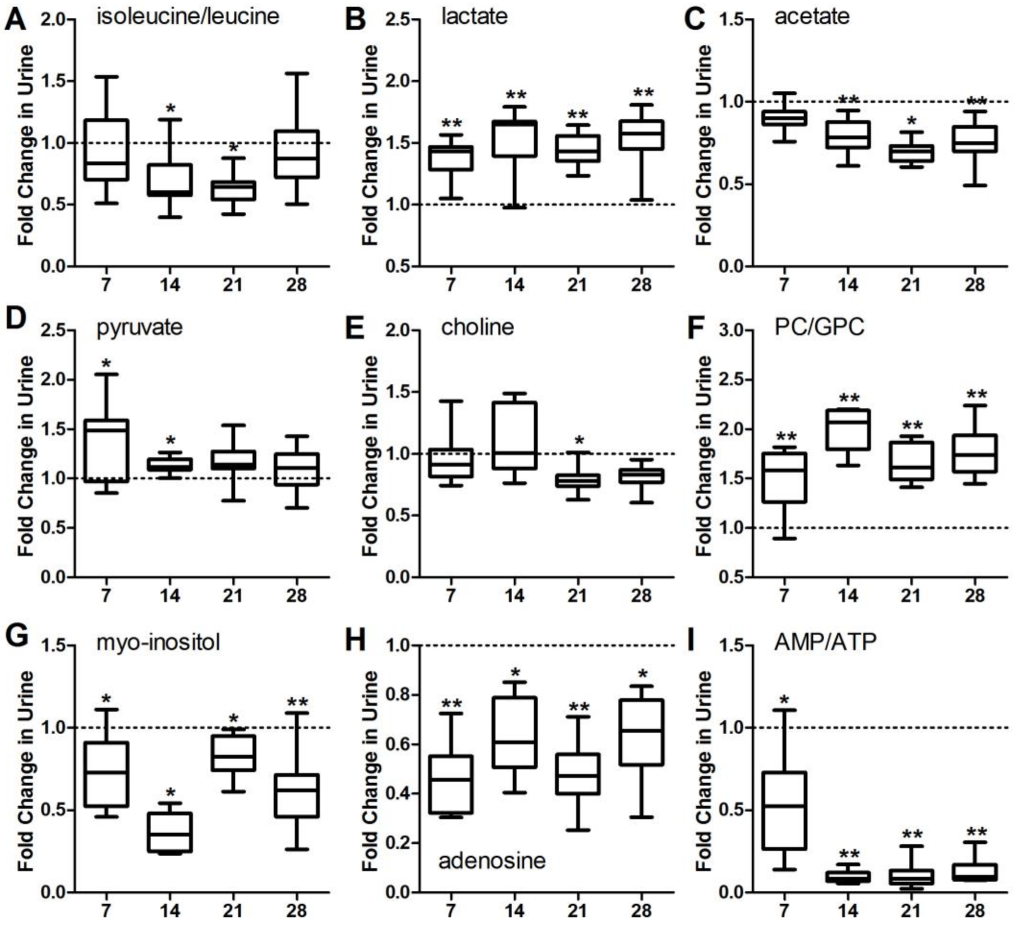

Figure S2 | Relative abundances of metabolites obtained from ^{1}H NMR spectra of kidney samples collected from the UUO rats compared to the age-matched control rats at the different time points after UUO or sham surgery. Keys: PC/GPC, phosphocholine/glycerophosphosphorylcholine.

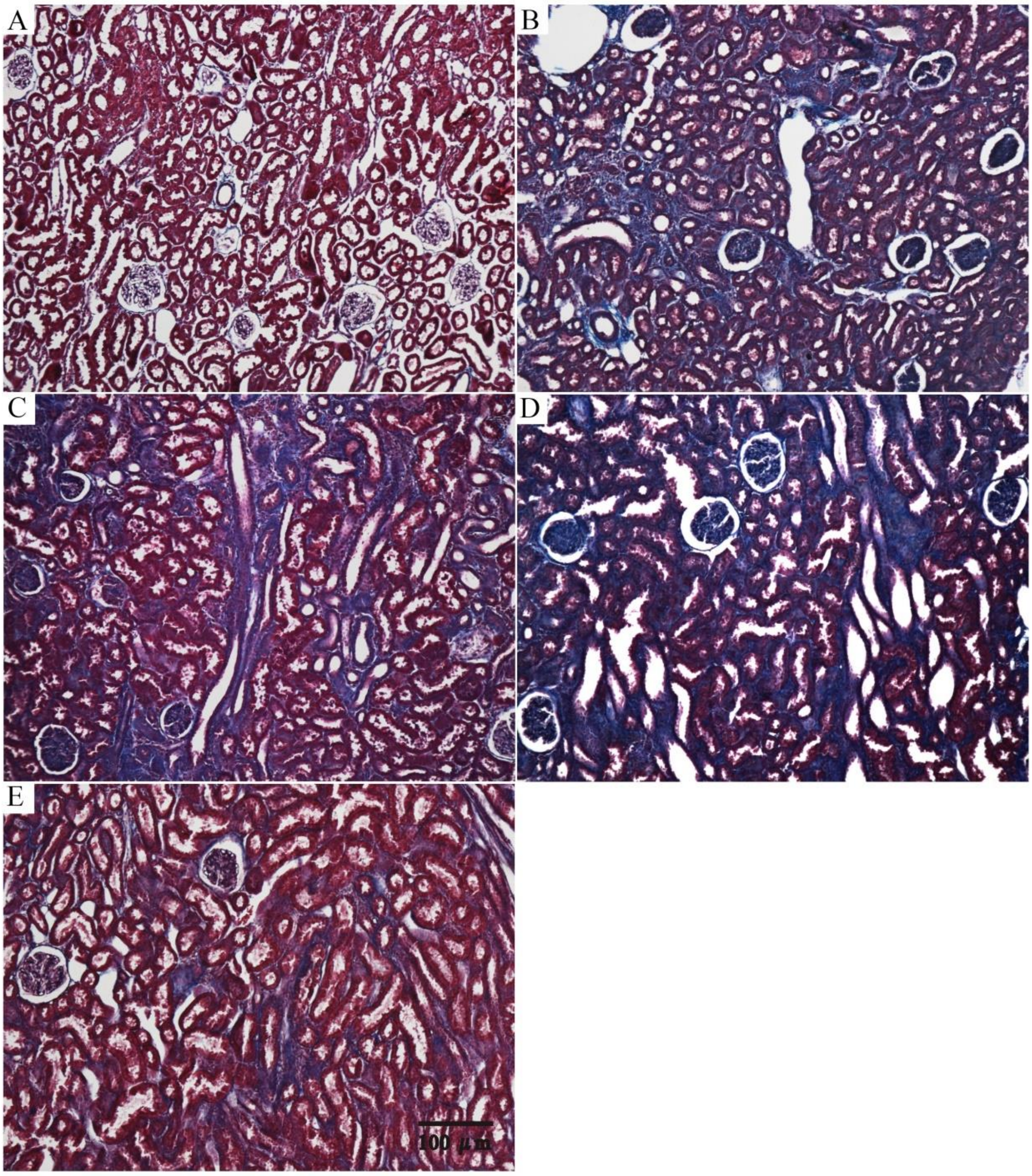

Figure S3 | Representative Masson-stained sections (100-fold) of kidneys from Sham-operated (A), UUO (B), and BCAAs-treated UUO rats (C), ATP-treated UUO rats (D), and meclofenamate-treated UUO rats (E) for 28 days, respectively.
